# The Use of Glass to Optimize Bitumen Absorption of Hot Mix Asphalt Containing Recycled Construction Aggregates

**DOI:** 10.3390/ma11071053

**Published:** 2018-06-21

**Authors:** Farzaneh Tahmoorian, Bijan Samali, John Yeaman, Russell Crabb

**Affiliations:** 1 Centre for Infrastructure Engineering, Western Sydney University, Penrith, NSW 2751, Australia; B.Samali@westernsydney.edu.au; 2 Faculty of Science, Health, Education and Engineering, University of Sunshine Coast, Sippy Downs, QLD 4556, Australia; johnyeaman@bigpond.com; 3 Asphalt, Boral Ltd., North Ryde, NSW 2113, Australia; russell.crabb@Boral.com.au

**Keywords:** asphalt, glass, recycled construction aggregate, volumetric properties, binder film index

## Abstract

Asphalt mixtures containing recycled construction aggregates (RCA) have the problem of high bitumen absorption. This paper characterizes the effects of glass on the bitumen absorption and volumetric properties of asphalt mixtures containing 25% and 50% RCA through laboratory investigation. The materials used in the test program include C320 bitumen, RCA and recycled glass. Three glass contents of 0%, 10%, and 20% in terms of the total weight of fine aggregates are used in the mixture designs for preparing 100 mm diameter specimens containing 0%, 25% and 50% RCA, under 120 gyration cycles. Different types of tests including aggregate specification tests and volumetric analysis tests were conducted on individual aggregates and asphalt mixtures in accordance with Australian standards. The test results indicate that the glass waste can be a viable material for improving the problem of high bitumen absorption of asphalt mixtures containing RCA.

## 1. Introduction 

Waste materials are generated increasingly with the continuous growth in the economy and as consumption increases. The growing quantities of waste materials, lack of natural resources and shortage of landfill spaces represent the importance of finding innovative ways of reusing and recycling waste materials. Due to the large quantities of construction and demolition waste (CDW), recycling and utilization of Recycled Construction Aggregates (RCA) obtained from CDW in construction projects, including asphalt pavement construction, can be the most promising solution to this problem. Utilization of RCA in asphalt mixtures is a sustainable technology due to the important role and high portion of aggregates in asphalt mixtures. In addition, RCA has better characteristics for Flakiness Index and Particle Shape compared to basalt [1]. Since these two characteristics substantially influence the stability and strength of asphalt mixture [2], they can be considered as one of the strong points of RCA. 

However, the major drawback of RCA is its high water absorption compared to conventional aggregates which subsequently results in high bitumen absorption of asphalt mixtures containing RCA. By combining materials with low absorption, such as glass, the high bitumen absorption of asphalt mixtures containing RCA can be compensated. Using waste glass in RCA-contained asphalt mixtures reduces not only bitumen absorption but also the adverse environmental impacts associated with waste glass disposal due to the nonmetallic and inorganic nature of glass waste, which makes it impossible to be disposed in incinerators or sanitary landfills. In addition, the demand reduction for virgin aggregates is another advantage resulting in subsequent economic advantages.

**Figure 1:**
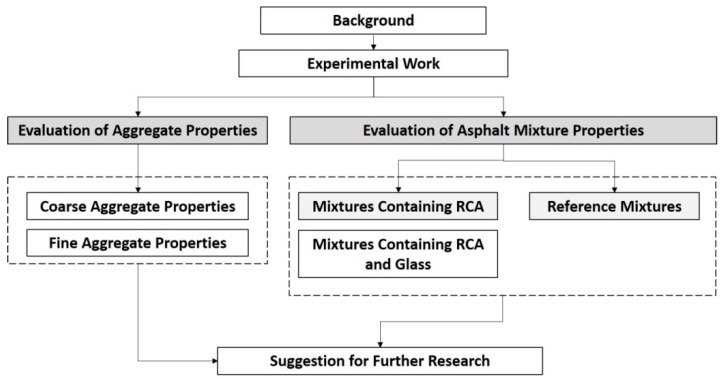
The flowchart of discussion in this research work.

However, developing a suitable mix design containing RCA and glass is necessary before employing this technology in asphalt mixture production. The purpose of the present work is to study the benefit of glass addition on the bitumen absorption of asphalt mixtures containing RCA in order to optimize the RCA-contained asphalt mix design. In that sense, as discussed in the following sections, several tests were conducted on individual aggregates in order to obtain further knowledge on the recycled aggregate properties as well as to compare them with the corresponding properties of the virgin aggregate and the standards requirements. Based on the results of the aggregate specification tests, different tests were conducted on asphalt mixtures containing various combinations of natural and recycled aggregates in order to investigate their performance in asphalt mixture and to characterize the effects of glass on the bitumen absorption and volumetric properties of asphalt mixtures containing different percentages of RCA. The findings of the experimental work are given in three main sections including the mechanical and physical properties of coarse aggregates (i.e., RCA and basalt), the physical characteristics of fine aggregates (i.e., glass and basalt) and volumetric performance of Hot Mix Asphalt (HMA) containing RCA in combination with glass and without glass. Figure 1 illustrates the flowchart of discussion in this research work.

## 2. Background

Today, many waste materials such as tyres, plastics, waste glass, etc. are used for construction of different layers of pavements including the asphalt surface layer [3,4,5,6,7,8,9,10,11,12,13,14]. Utilization of solid wastes in the asphalt layer not only reduces the adverse impacts of waste disposal but also the demand for natural materials which will subsequently results in cost savings and economic advantages. Moreover, using the recycled materials in the asphalt surface layer can contribute to more improvement of engineering characteristics of the asphalt pavement materials, representing a value-added application for solid wastes. However, the selection of waste materials used for road construction, particularly the surface course, is of high importance as the incorporation of wastes should not affect the functional and structural aspects of the pavements [15,16,17].

In addition, due to the importance of the aggregates in asphalt concrete, the studies on the utilization of the recycled aggregates such as reclaimed asphalt pavement (RAP), recycled construction aggregate (RCA), recycled glass, etc. have increased worldwide over the past two decades [18,19,20]. Among the recycled aggregates, the large amount of construction and demolition waste generation worldwide justifies the idea of using RCA in new asphalt mixtures. Referring to available literature e.g., [21,22,23,24,25,26,27,28], RCA has been used in the base course and subbase course of pavements over the last two decades. However, few research studies [29,30,31,32,33,34,35,36] have reported the utilization of RCA in HMA.

According to comprehensive aggregate specification tests on RCA, RCA cannot satisfy aggregate requirements for asphalt mixtures for two properties of water absorption and wet/dry strength variation [37]. Accordingly, the application of RCA without any virgin aggregates may result in asphalt mixtures with less efficiency. Therefore, it is required to consider the combination of RCA with other aggregates in certain percentages for the asphalt mixture design. Accordingly, some other materials such as recycled glass can be considered to compensate RCA for some of its shortcomings, and this research will seek for the optimum combination of these materials. 

In Australia, about 850,000 tonnes of glass are consumed, of which only 350,000 tonnes are recycled [38]. This means that approximately 500,000 tonnes of glass are disposed into the landfill every year. The Australian example is indicative of the glass waste disposal throughout the world. 

Using waste glass in combination with RCA in asphalt mixture provides substantial environmental and economic advantages since glass waste is one of the most important and particularly troublesome components of solid waste because it cannot be incinerated or degraded. Therefore, it is required to consider a proper approach for its management. Recycling is the most common method for handling glass wastes. In fact, glass can be recycled without any loss in the product quality. Recycling the glass wastes will result in substantial savings of energy as well as mineral resources. In addition, the recycling of glass helps to alleviate the increasing cost of landfill disposal [39]. However, the variations in glass colour have encouraged the authorities to seek alternative approaches to glass waste management.

Many countries such as the United States, Japan, and several European countries have used glass waste as a substitution for fine aggregate in asphalt mixtures [40]. However, glass as aggregate in asphalt concrete should meet some technical specifications. Accordingly, many researchers [41,42,43] have studied the utilization of glass as aggregate for asphalt mixtures. Arabani and Azarhoosh (2011) studied the behaviour of asphalt mixtures containing glass (glassphalt) at different temperatures and by using different sizes of glass at different rates. The results of this investigation revealed that adding glass will improve the dynamic behaviour of and the stiffness of asphalt mixtures. In addition, asphalt mixtures containing glass have less temperature sensitivity compared to conventional mixtures [44]. In another study by Jony et al. (2011), the effect of utilizing different fillers (including glass powder) at different rates in asphalt mixtures was investigated. The results of this investigation indicated that using glass powder as filler improves the Marshall Stability of asphalt mixtures in comparison with the asphalt mixtures made with Portland cement or limestone powder as filler [45]. Pereira et al. (2010) conducted research on the utilization of waste flat glass as filler in asphalt mixtures. This research concluded that waste glass can be effectively used as filler in asphalt mixtures. In another field study, two sections of road using two sizes of crushed glass were constructed in Minnesota [46]. Referring to Marti et al. (2002), the results of a rutting test on these roads revealed that the incorporation of waste glass with a size of 9.5 mm in asphalt mixtures provides asphalt mixtures with less dynamic stability compared to asphalt mixtures containing waste glass with a maximum size of 4.75 mm [47]. 

Other research by Arnold et al. (2008) showed that the addition of up to 30% glass waste by mass of aggregates will not significantly change the aggregates performance [48]. Shafabakhsh and Sajed (2014) concluded that asphalt mixtures containing 10 to 15% crushed glass perform satisfactorily [49]. Finkle and Ksaibati (2007) reported that waste glass can be used as an alternative to the virgin road base materials. However, the glass content of up to 20% and maximum size of 12 mm was recommended based on this research [50]. Wu et al. (2013) investigated the performance of asphalt mixtures containing waste glass as fine aggregate. The maximum size of 4.75 mm and the optimum content of 10% were recommended by this research [34]. In a report published by Australian Road Research Board (ARRB) Group for the Packaging Stewardship Forum (PSF) of the Australian food and grocery council (2012), the glass content of up to 20% was recommended for the utilization in asphalt mixtures as fine aggregate. This report limits the utilization of waste glass to 30% by mass of the total fine aggregate in asphalt mixture [51].

Referring to Su and Chen (2002), in a research program in Taiwan, the engineering properties of the asphalt mixtures incorporating the crushed glass waste were studied through the laboratory and field tests. The result of this research revealed that the utilization of glass waste in asphalt mixtures provides substantial economical and engineering advantages [52]. Pioneer Road Services carried out the first glass mix trials in Australia in 2003. The roads were compared with conventional asphalt roads for skid resistance properties. The investigation showed that the skid resistance of the asphalt mixtures containing glass waste is similar to conventional asphalt mixtures [53]. Based on research by Viswanathan (1996), the waste glass can be used in highway construction [54]. 

In general, glass is typically brittle and has very low impact resistance. This physical property of glass has been used positively in crushing the waste glass in desirable sizes with low energy consumption. Furthermore, glass shows high volumetric stability under high temperatures of up to 700 °C. The thermal expansion coefficient and softening point of glass are in the range of 8.8 to 9.2 × 10^−6^ cm/°C and 718 to 738 °C, respectively.

According to available literature, it can be concluded that the application of glass in asphalt mixtures has both advantages and disadvantages, as summarized in Table 1, which introduces some limits on the utilization of glass in asphalt mixture as follows:

- The use of recycled glass is recommended to be limited to 20% as the maximum replacement rate in asphalt mixture
- In case of glass content of more than 15% of the total mixture, it is required to add 1 to 2% antistripping agent to the asphalt mixture in order to avoid the stripping problems. Hydrated lime is an effective antistripping agent which can be used in asphalt mixtures containing glass.
- Suitable particle size of glass as aggregates in asphalt mixture is 4.75 mm or smaller.

Through this information, it can be easily understood that the application of waste materials in asphalt mixtures directly affects the behaviour of the asphalt mixtures, leading to both advantages and disadvantages of overall asphalt mixture performance. Recognizing this fact, having knowledge about the properties of individual components in asphalt mixtures and their combination will result in selecting the best combination of aggregates for designing an optimum asphalt mixture. 

## 3. Experimental Work

### 3.1. Materials

In the present study, RCA, glass and basalt have been used as aggregates and the original bitumen studied in this research corresponds to C320, which is the most common binder for wearing courses subjected to heavy loading and/or in hot climates. The typical characteristics of Bitumen C320 are presented in Table 2.

The virgin aggregate (basalt) was obtained from Nepean Quarries which is a local quarry in Sydney. RCA was obtained from the Revesby Recycling Centre located in Revesby, NSW, Australia. This centre is a transfer station accepting residential and commercial wastes. Based on a statistical study on RCA samples collected over one year, it was observed that there are different construction wastes in RCA, which is mostly (about 64%) composed of sandstone or an agglomerate of sand and cement paste. In addition, a matrix of portland cement concrete will vary between basalt (i.e., Basic Igneous) and granite (i.e., Acidic Igneous) depending on the source of material and the age of the building from which it came makes 17% of RCA. Also, ceramic, glass and brick make about 19% of RCA. The result of the statistical study on RCA is reported in a separate paper.

Recycled glass used in this research was clear crushed glass made from recycled glass and passed through 4.75 mm sieve size and was obtained from Schneppa Glass (Burwood, VIC, Australia). 

The fillers considered in this research are hydrated lime and Portland cement. Using the correct amount of hydrated lime, approximately 2% by weight, in mix designs will improve the durability of mixtures and will minimize the problem of stripping, particularly in asphalt mixtures made with partial glass substitution.

### 3.2. Laboratory Tests on Coarse Aggregates

As presented in the previous sections, in this research project, attempts are made to evaluate the suitability of RCA as part of coarse aggregate in asphalt mixture. Since the basic properties of materials are essential factors in any asphalt mixture design, the fundamental properties of RCA are investigated through conducting different specification tests on different coarse aggregates used in this research (i.e., RCA and basalt). 

### 3.3. Laboratory Tests on Fine Aggregates

To achieve the goals of this research, the study of properties of recycled glass as part of fine aggregate in combination with basalt has been considered as part of this research work. In light of this, different tests have been conducted on recycled glass and basalt (passed 4.75 mm sieve size). 

### 3.4. Laboratory Tests on Asphalt Mixtures Containing Recycled Materials 

#### 3.4.1. Sample Preparation

In this study, the asphalt specimens were made from materials mixed in the Centre for Infrastructure Engineering (CIE) laboratory at Western Sydney University. About 4 kg of materials were used to produce three batches of laboratory asphalt mixtures for a finished specimen of diameter 100 ± 2 mm and height of 65 ± 5 mm, in accordance with AS2891.2.1 [60] and AS2891.2.2 [61] using an IPC gyratory compactor.

For this experimental work, a group of specimens were prepared without recycled materials (0%) as reference to specimens made with 25% and 50% RCA and 0% glass substitution. In addition, in order to assess the effect of glass on the bitumen absorption of asphalt mixtures containing RCA, two groups of specimens were also prepared with 25% and 50% RCA and glass at the rates of 10% and 20%. The glass substitution was made on each sieve from #4 down to #8. Therefore, 66 samples of the following asphalt mixtures were prepared for this study, as indicated in Table 3.

**Figure 2:**
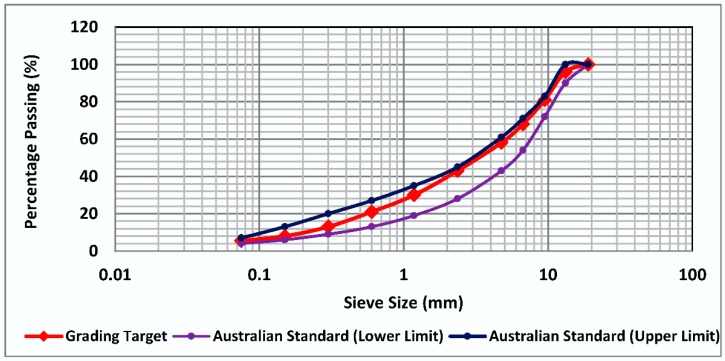
Gradation curve of asphalt mixtures.

Figure 2 illustrates the design gradation curve used for the preparation of mixtures. It should be noted that portland cement and hydrated lime were used in all asphalt mixtures as filler (passing 0.075 mm sieve). 

#### 3.4.2. Evaluation of Volumetric Properties of Asphalt Mixtures 

It is generally recognized that the volumetric composition of mixtures greatly influence their performance. The volumetric properties evaluation of asphalt mixtures is the fundamental of asphalt mix design, determining the performance of asphalt mixture. The asphalt mixture volumetric properties including void content, voids filled with binder (VFB) and voids in mineral aggregate (VMA) have been recognized as important parameters affecting the durability and performance of asphalt pavements [62]. The minimum values are typically required for volumetric parameters depending on the asphalt mixture type. 

As mentioned previously, all asphalt mixes in this research are dense-graded asphalt (DGA) with a nominal size of 14 (AC14), which are prepared in accordance with Test Method RMS T661 [63] and RMS T662 [64] (120 cycles of compaction) which are identical to AS2891.2.1 [60] and AS2891.2.2 [61], respectively. The requirements for volumetric parameters of this type of mixture are summarized in Table 4. In addition, a summary of the tests carried out to study the mixtures properties is explained in the following sections.

##### Bulk Density Test

In this research, the bulk density of compacted samples is determined using the presaturation procedure in accordance with AS/NZS 2891.9.2 [65]. This method is suitable for dense-graded mixtures with internal air voids that are largely inaccessible to moisture resulting in low permeability. 

##### Maximum Density Test

In this study, the maximum density of a loose sample of mix is determined using the methylated spirits displacement procedure, in accordance with AS/NZS 2891.7.3 [66]. Based on this test method, firstly, the density of methylated spirit ($\rho_{m}$) was determined as 0.789 t/m^3^. 

##### Voids and Volumetric Properties

The voids and volumetric properties of asphalt mixtures are determined, in this research, in accordance with AS/NZS 2891.8 [67].

#### 3.4.3. Evaluation of Resilient Modulus of Asphalt Mixtures 

The stiffness of asphalt mixtures is a fundamental property and plays an important role in determining the performance of asphalt pavement under traffic loading. The resilient modulus is the measure of stiffness of asphalt mixtures. In addition, the resilient modulus of asphalt mixtures is useful in determination of layer thickness through estimation of the relative strength coefficient and calculation of Structural Number (SN). 

In this study, resilient modulus test was considered for evaluation of the stiffness of some specimens selected based on the results of a primary test to assess the effect of the RCA amount as well as the asphalt mixture composition on resilient modulus. To this point, the asphalt mixtures were prepared using different combinations but with the same gradation. Subsequently, the asphalt mixtures were compacted with GyroPac at the same level of compaction to make cylindrical specimens of 100 mm in diameter and 65 mm in height. 

The resilient modulus, in this research, was determined through an indirect tensile strength test in accordance with AS/NZS 2891.13.1 [68]. In this test, firstly, the diameter and height of specimens were measured. The specimen was placed in the temperature-controlled cabinet at the temperature of 25 °C to allow the temperature in the specimen to reach equilibrium prior to the test. Then, the machine and Linear Variable Differential Transformers (LVDT) were calibrated to conduct the test. 

**Figure 3:**
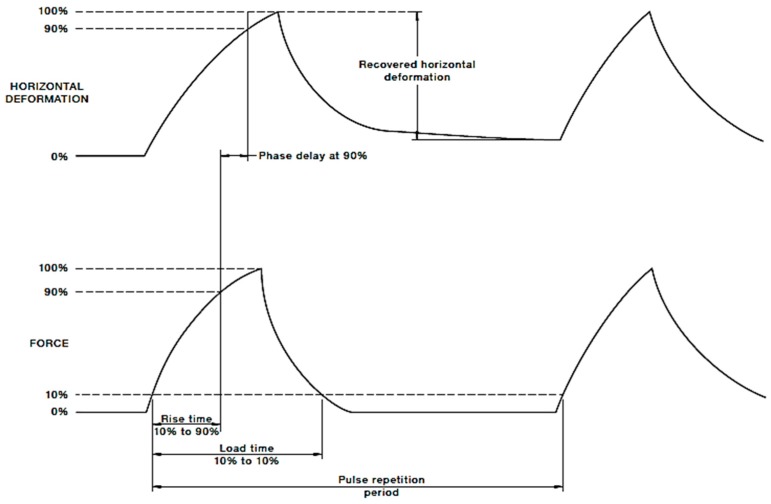
Load and horizontal deformation graph in Resilient Modulus Test.

During the test, repeated haversine loading is applied to the specimen at the frequency of 0.1 Hz considering 0.1 s loading and 0.9 s rest period (Figure 3).

Following the preconditioning, five load pulses were applied with a certain rise time to the peak load at a certain pulse repetition period. The recovered horizontal deformation of the specimen after application of each load pulse was recorded. The Poisson’s ratio is considered as 0.4 in accordance with AS 2891.13.1 [68]. The resilient modulus $\left(M_{r}\right)$ in MPa for each specimen for each load pulse during the resilient modulus test were obtained from the following equation:

(1) $$M_{r}=P\times \frac{\left(\mu +0.27\right)}{H\times h_{c}}$$

where P is peak load (N), μ is Poisson ratio, H is recovered horizontal deformation of the specimen after load pulse (mm), and $h_{c}$ is the height of specimen (mm).

## 4. Results and Discussion 

### 4.1. Coarse Aggregate Tests Analysis

The properties of RCA and basalt were investigated throughout a comprehensive experimental work. The results of these tests on three samples for each aggregate type are summarized in Table 5. 

As can be observed in Table 5, all properties of RCA, except for water absorption and wet strength, meet the Australian Standards’ requirements, and hence, further investigation on the feasibility of the utilization of RCA as part of basalt in asphalt mixtures appears plausible.

Importantly, RCA displays a smaller value for two parameters of Flakiness Index and Particle Shape in comparison with basalt. These two parameters significantly affect the final performance of asphalt mixtures, and better values for these properties can be one of the strong points of RCA contributing to the improvements in compaction, rutting resistance, and workability of asphalt mixtures.

In addition, as can be seen in Table 5, the results indicate that RCA has substantially higher absorption in comparison to basalt, mainly due to the presence of great amounts of impurities and cracks in RCA. The water absorption of RCA is also more than the typical limit specified in the Australian Standard. Since the high water absorption of RCA may result in high bitumen absorption of asphalt mixtures, the necessity of finding and studying potential materials to compensate for this problem of RCA has led to the idea of utilization of glass waste in combination with RCA in asphalt mixture design, which is the main goal of this paper.

### 4.2. Fine Aggregate Tests Analysis

As discussed in the previous sections, the recycled glass is used as part of fine aggregates in this research to compensate for high absorption of RCA. Hence, some of the properties of glass and basalt were studied through conducting a series of tests. These tests and their results analysis are summarized in Table 6. 

The data given in Table 4 clearly displays the low amount of water absorption of glass in comparison with fine basalt and Australian standard limits, which makes it an adequate option for combination with RCA.

### 4.3. Volumetric Analysis of Asphalt Mixtures Containing RCA

The volumetric properties of the basalt-RCA asphalt mixtures were determined and then compared with the standards specifications. According to Austroads (2014), the essential parameters in the level 1 of mix design include air voids, voids in mineral aggregate (VMA), and voids filled with binder (VFB), [76]. 

Table 7 presents the properties obtained for asphalt mixtures with different bitumen content and aggregate combination at a selected level of gyrations (120 cycles).

#### 4.3.1. Optimum Bitumen Content Determination 

**Figure 4:**
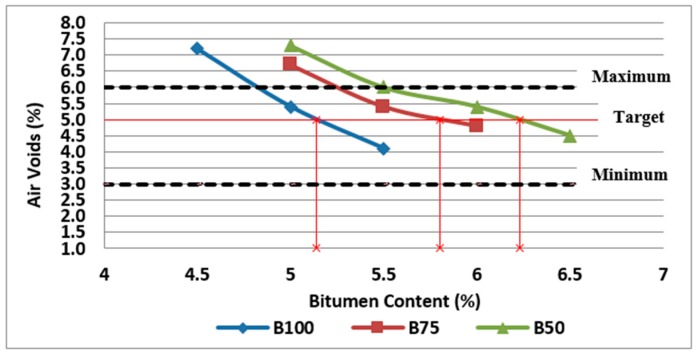
Effect of bitumen content and RCA content on air voids of Mix I containing virgin aggregate and Mix II and Mix III containing RCA as coarse aggregate.

To determine the optimum bitumen content for the asphalt mixture, the procedure indicated by Australian standards, AGPT04B-14, was followed in this research. Three specimens at each bitumen content (4.5%, 5%, 5.5%, 6%, and 6.5%) were tested for maximum density, bulk density, and subsequently air voids and VMA calculations. The results of these tests and calculations are used to determine the optimum bitumen content that provides air voids within the specified limits and VMA near to the minimum value. According to the results obtained, Figure 4 illustrates the effect of bitumen content and RCA content on air voids of asphalt mixtures. 

The air void content in the mix is a function of bitumen content, degree of compaction and VMA. The air void percentage in the mixture affects mix stiffness, fatigue resistance, and durability. As shown in this figure, air voids decrease with the bitumen content increase. Air voids of mixtures made with RCA are substantially higher than the control mixtures because of porous cement paste attached to the virgin aggregates and also porous structure of some aggregates in the RCA.

Generally, asphalt mixtures should have the lowest practical air voids in order to reduce the binder ageing and the permeability and subsequent stripping problems. However, referring to Austroads (2014), plastic flow and subsequent bleeding, flushing, shoving or permanent deformation of the pavement may occur if the air voids are too low (less than about 2%). Accordingly, as can be observed in Figure 4, some of the mixtures (i.e., B100-4.5, B75-5 and B50-5) cannot be acceptable in terms of air voids requirements. As discussed previously, the optimum bitumen content can be obtained for design air voids of 5%. Based on the results, the optimum bitumen content was found to be 5.1% for reference samples (0% RCA), 5.8% for samples with 25% RCA and 6.2% for samples with 50% RCA, as illustrated in Figure 4. 

Furthermore, VMA is another important volumetric property which should be checked for selection of the final bitumen content. VMA is the combination of air voids in the compacted mix and the volume occupied by the effective binder which is total binder minus any binder absorbed into the aggregate. VMA is a function of the gradation and the particle shape and surface texture of the aggregate particles. VMA should be large enough to provide a sufficient amount of air voids in the compacted mixture for ensuring the asphalt mixture stability while leaving enough space for the binder to ensure the mixture durability. If VMA is too low, the binder would be insufficient for cohesion and durability whereas too high VMA results in more costly asphalt mixtures due to increased binder volume to satisfy the air voids requirements.

**Figure 5:**
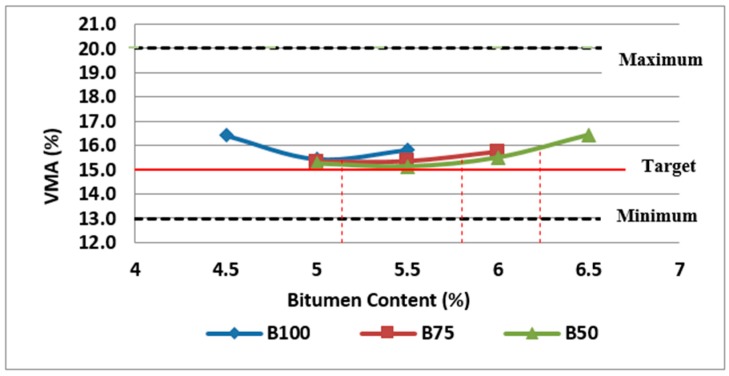
Effect of bitumen content and RCA content on VMA of Mix I containing virgin aggregate and Mix II and Mix III containing RCA as coarse aggregate.

The variation of VMA with bitumen content for Mix I, Mix II and Mix III are shown in Figure 5. As can be observed in Figure 5, VMA increases with bitumen content after a minimum point. The test results on different samples showed that VMA of mixtures containing RCA is quite a bit lower than the control samples which can be as a result of higher bitumen absorption of RCA resulting in the lower amount of not-absorbed binder (effective binder). As illustrated in Figure 5, mixtures at optimum bitumen content meet the requirements of 15% (minimum) for VMA.

#### 4.3.2. Determination of Bulk Density and Water Absorption

**Figure 6:**
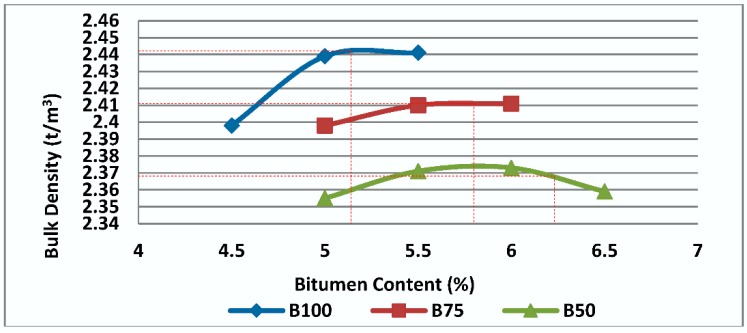
Effect of bitumen content and RCA content on bulk density of Mix I containing virgin aggregate and Mix II and Mix III containing RCA as coarse aggregate.

Bulk density is an important parameter used for volumetric properties evaluation of asphalt mixtures. Bulk density of Mix I, Mix II and Mix III are shown in Figure 6. It can be seen that bulk densities of mixtures containing RCA are considerably lower than bulk density of mixtures made with virgin aggregates (Mix I), mainly because of the low density of cement paste and RCA particles.

**Figure 7:**
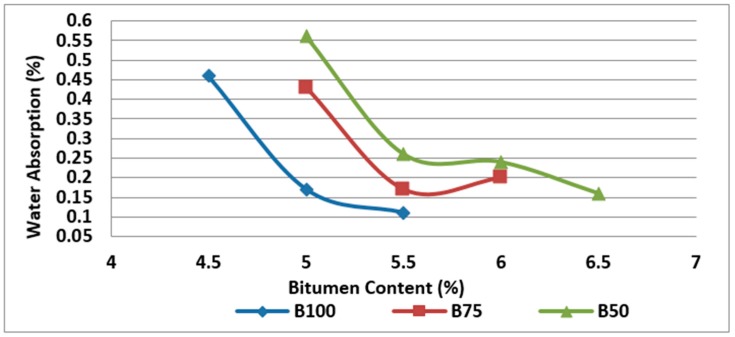
Effect of bitumen content and RCA content on water absorption of Mix I containing virgin aggregate and Mix II and Mix III containing RCA as coarse aggregate.

Furthermore, the experimental results show the water absorption increase of the mixtures by the increase in amount of RCA at the same bitumen content due to porous structure of RCA, as expected and shown in Figure 7.

#### 4.3.3. Determination of Voids Filled with Bitumen (VFB)

Another important volumetric parameter is voids filled with binder (VFB). VFB is defined as the ratio of the effective binder (by volume) and the VMA. Mixtures with low VFB are dry and lack durability, cohesion and fatigue resistance. 

**Figure 8:**
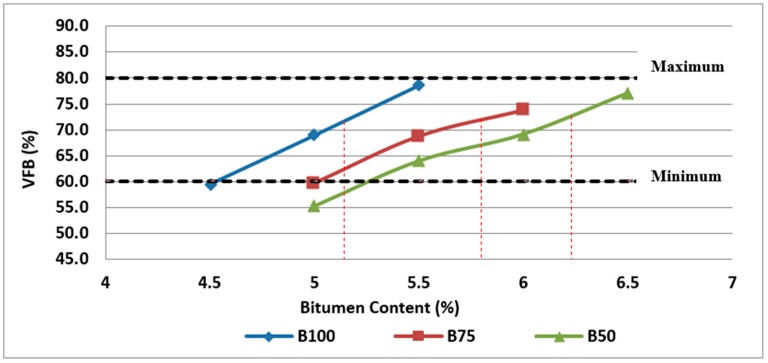
Effect of bitumen content and RCA content on VFB of Mix I containing virgin aggregate and Mix II and Mix III containing RCA as coarse aggregate.

These mixtures may also be more permeable, whereas asphalt mixtures with too high VFB can become unstable and susceptible to rutting. Figure 8 illustrates the variation of VFB with the RCA and bitumen content. The VFB values obtained for asphalt mixtures containing RCA are relatively lower compared to the control samples because of higher absorption of RCA leading to a less amount of not-absorbed binder (effective binder).

#### 4.3.4. Determination of Binder Film Index (BFI)

**Figure 9:**
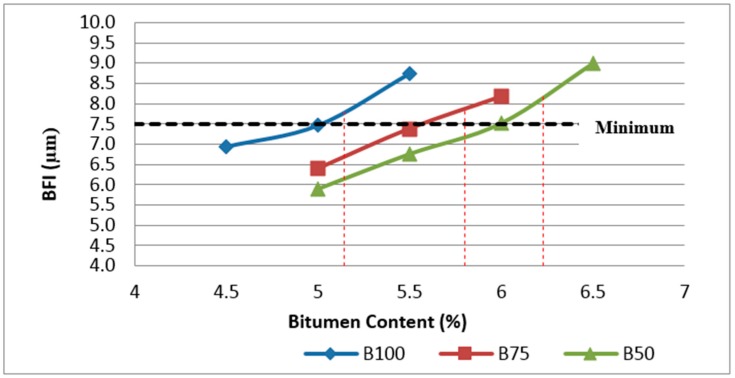
Effect of bitumen content and RCA content on BFI of Mix I containing virgin aggregate and Mix II and Mix III containing RCA as coarse aggregate.

Binder Film Index (BFI) is another parameter that can be considered at the volumetric design stage as a guide to the incorporation of sufficient binder in the asphalt mixture to ensure adequate durability, cohesion, resistance to the effects of moisture and fatigue resistance (Austroads, 2014). BFI is a function of the surface area of filler and the aggregates as well as the effective bitumen content. According to the results obtained, the binder film index of Mix I, Mix II and Mix III are shown in Figure 9. As can be observed in Figure 9, BFI values for Mix II and Mix III are lower than BFI for control samples (Mix I) at the same bitumen content, as more binder is absorbed by the mixtures incorporating RCA, resulting in less aggregate particles coating due to the reduction of available binder for this purpose. As can be expected, the BFI is increased with the increase in bitumen content. 

In addition, as can be seen in Figure 9, all samples at their optimum bitumen content meet the minimum requirements of 7.5 µm for BFI. 

### 4.4. Volumetric Analysis of Asphalt Mixtures Containing RCA and Glass

Since asphalt mixtures made with RCA have the problem of high absorption, as discussed in previous sections, it is desired to optimize the absorption characteristics of these asphalt mixtures by adding recycled glass, which is the primary objective of this research work. 

To this end, different asphalt mixtures containing RCA with three glass contents of 0%, 10% and 20% (by weight of fine aggregates) were prepared for evaluating the effect of the addition of glass to RCA-basalt asphalt mixtures. For this purpose, different combinations of aggregates at different rates of bitumen content of 5%, 5.5% and 6% were considered to make specimens with 100 mm diameter at a required level of gyration (120 cycles) for the considered traffic category. Table 8 presents the results of volumetric analysis for asphalt mixtures containing RCA and glass with different bitumen content.

#### 4.4.1. Determination of optimum bitumen content for asphalt mixtures with glass

Similar to the procedure explained in Section 4.3.1, the optimum bitumen content for the asphalt mixtures made with RCA and glass were determined in accordance with Australian standards. To this end and in order to compare the effect of glass addition to RCA-basalt mixtures, three specimens at different bitumen contents of 5%, 5.5% and 6% made with 25% RCA and recycled glass at rates of 10% and 20% were prepared and tested for maximum density, bulk density, and subsequently air voids and VMA calculations.

The same bitumen content and glass content was also considered for preparation of samples containing 50% RCA. 

**Figure 10:**
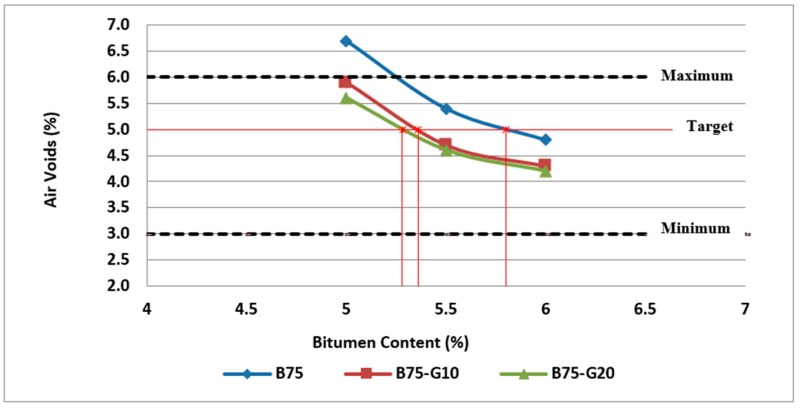
Effect of bitumen content and glass content on air voids of Mix II containing 25% RCA without glass and Mix IV and Mix V containing 25% RCA as coarse aggregate and glass as fine aggregate.

**Figure 11:**
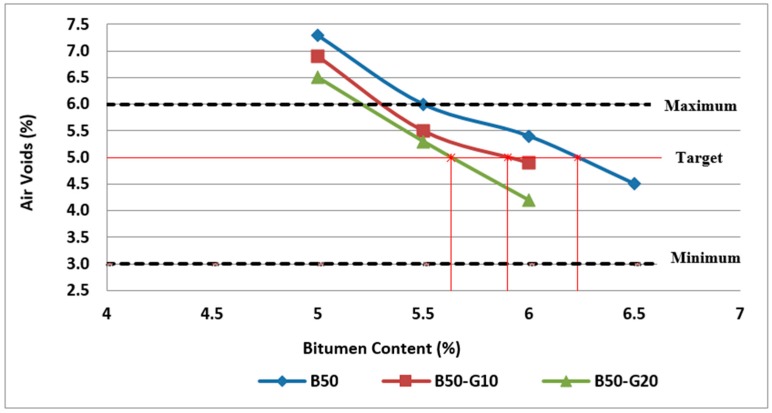
Effect of bitumen content and glass content on air voids of Mix III containing 50% RCA without glass and Mix VI and Mix VII containing 50% RCA as coarse aggregate and glass as fine aggregate.

The effect of bitumen content and glass content on air voids of asphalt mixtures is illustrated in Figure 10 and Figure 11. As can be clearly observed in Figure 10 and Figure 11, air voids of mixtures containing glass are lower than the mixtures containing RCA without glass due to highly hydrophobic property of glass. In addition, air voids decrease with the increase of bitumen content in all samples. 

**Figure 12:**
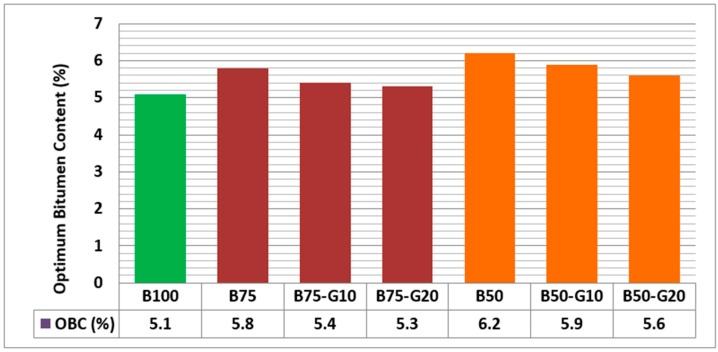
Optimum bitumen content (OBC) of asphalt mixtures.

Importantly, the results of volumetric analysis and air void calculations based on the bulk density test and maximum density test reveal that the optimum bitumen content of asphalt mixtures varies with the amount of waste glass used, so that mixtures containing more glass require less bitumen, as presented in Figure 10 and Figure 11. The results of the obtained optimum bitumen content based on all specimens studied in this research work are presented in Figure 12.

**Figure 13:**
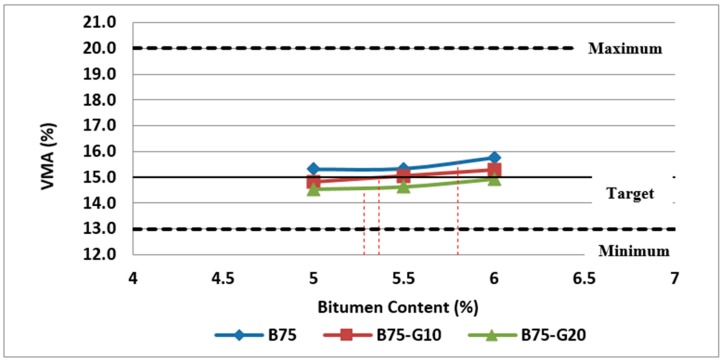
Effect of bitumen content and glass content on VMA of Mix II containing 25% RCA without glass and Mix IV and Mix V containing 25% RCA as coarse aggregate and glass as fine aggregate.

**Figure 14:**
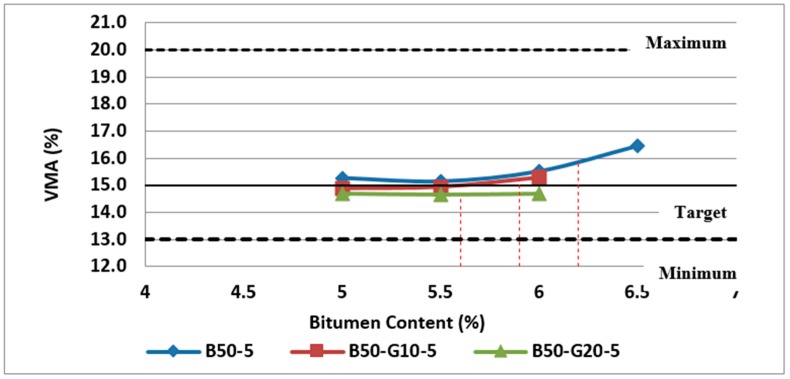
Effect of bitumen content and glass content on VMA of Mix III containing 50% RCA without glass and Mix VI and Mix VII containing 50% RCA as coarse aggregate and glass as fine aggregate.

Furthermore, as discussed previously, VMA is another parameter required to be considered in selecting the optimum bitumen content. The variation of VMA with bitumen content for mixtures containing 25% and 50% RCA made with recycled glass or without glass are illustrated in Figure 13 and Figure 14, respectively. 

The test results on different samples showed that VMA of mixtures containing glass is quite a bit lower than the samples made with RCA without glass. It can be due to the lower air voids and lower particle density of combined mineral aggregates in samples made with RCA and glass. However, in all group of Mixes, VMA increases with bitumen content after a minimum point. 

As illustrated in Figure 13 and Figure 14, mixtures without glass and containing 10% glass meet the requirements of 15% (minimum) for VMA at their optimum bitumen content. However, VMA for other mixtures containing 20% glass is still in the acceptable range.

#### 4.4.2. Determination of Bulk Density and Water Absorption for Asphalt Mixtures with Glass

Bulk density test was conducted on specimens containing glass and RCA to measure the bulk density and water absorption of samples. 

**Figure 15:**
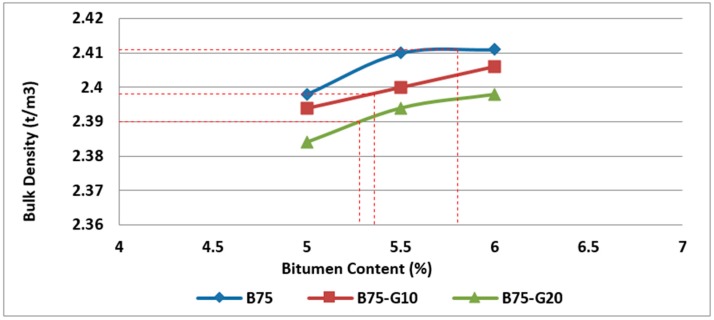
Effect of bitumen content and glass content on bulk density of Mix II containing 25% RCA without glass and Mix IV and Mix V containing 25% RCA as coarse aggregate and glass as fine aggregate.

**Figure 16:**
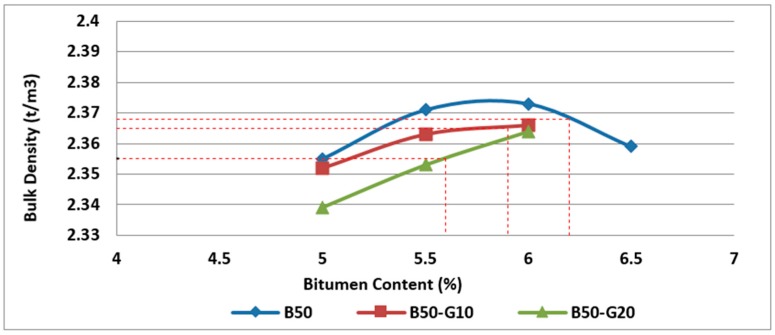
Effect of bitumen content and glass content on bulk density of Mix III containing 50% RCA without glass and Mix VI and Mix VII containing 50% RCA as coarse aggregate and glass as fine aggregate.

Based on the results obtained from the bulk density test on two groups of asphalt mixtures with 25% and 50% RCA in combination with different rates of recycled glass, it can be noticed that the bulk density decreases with the increase in the glass content due to lower bulk density of glass in comparison with basalt, as illustrated in Figure 15 and Figure 16.

**Figure 17:**
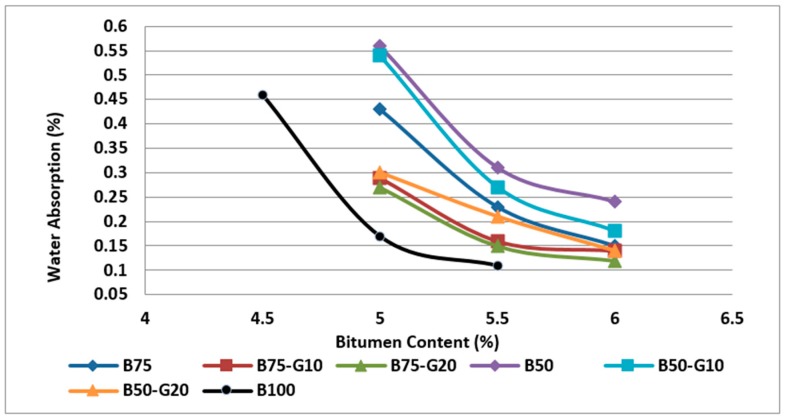
Comparison of water absorption in asphalt mixtures with different aggregate type.

In addition, the data obtained from the bulk density test indicate that water absorption decreases with respect to the amount of recycled glass, and asphalt mixtures containing glass appear to have low water absorption because of the hydrophobic property of glass. Figure 17 illustrates the water absorption of all different specimens. 

#### 4.4.3. Determination of Voids Filled with Bitumen (VFB) for Asphalt Mixtures with Glass

**Figure 18:**
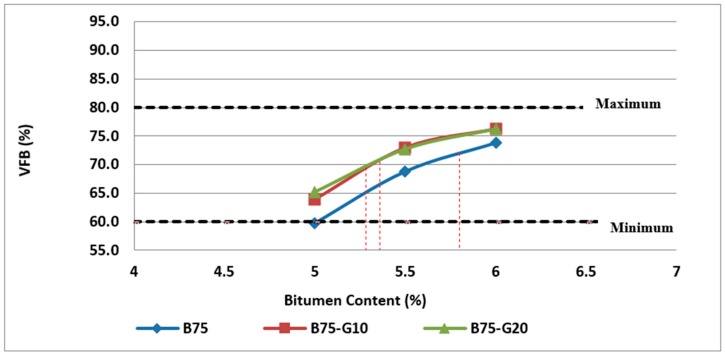
Effect of bitumen content and glass content on VFB of Mix II containing 25% RCA without glass and Mix IV and Mix V containing 25% RCA as coarse aggregate and glass as fine aggregate.

**Figure 19:**
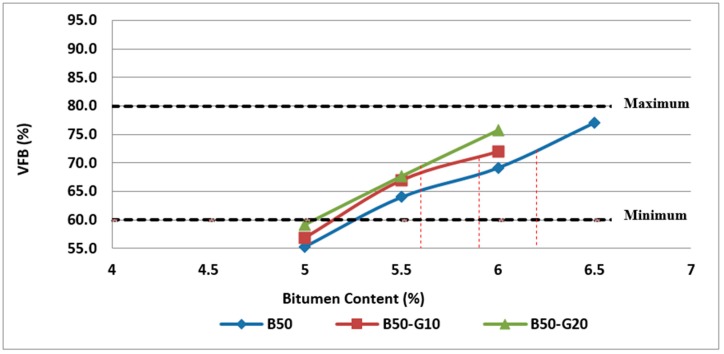
Effect of bitumen content and glass content on VFB of Mix III containing 50% RCA without glass and Mix VI and Mix VII containing 50% RCA as coarse aggregate and glass as fine aggregate.

Figure 18 and Figure 19 illustrate the variation of VFB with the bitumen content and glass content. As previously stated, VFB is one of the indicators of asphalt mixture performance in terms of durability, fatigue resistance and susceptibility to rutting.

As can be observed in Figure 18 and Figure 19, the values obtained for asphalt mixtures made with glass and RCA are higher than the samples containing RCA without glass due to a lower degree of absorption resulting in increased effective binder.

#### 4.4.4. Determination of Binder Film Index (BFI) for Asphalt Mixtures with Glass

As discussed previously, BFI is an indicator of adequate cohesion and the incorporation of sufficient binder in the asphalt mixture. 

**Figure 20:**
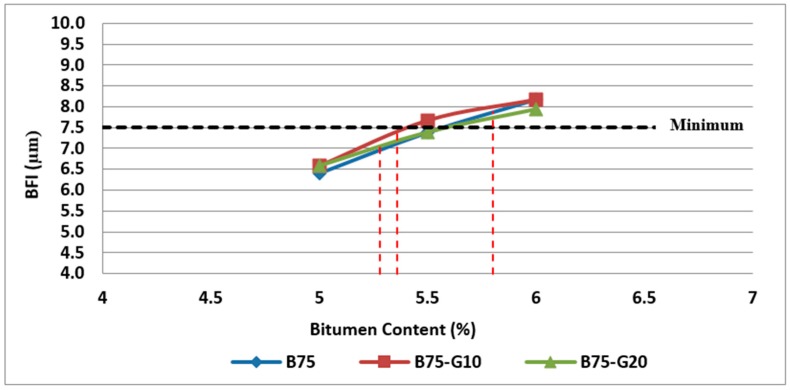
Effect of bitumen content and glass content on BFI of Mix II containing 25% RCA without glass and Mix IV and Mix V containing 25% RCA as coarse aggregate and glass as fine aggregate.

**Figure 21:**
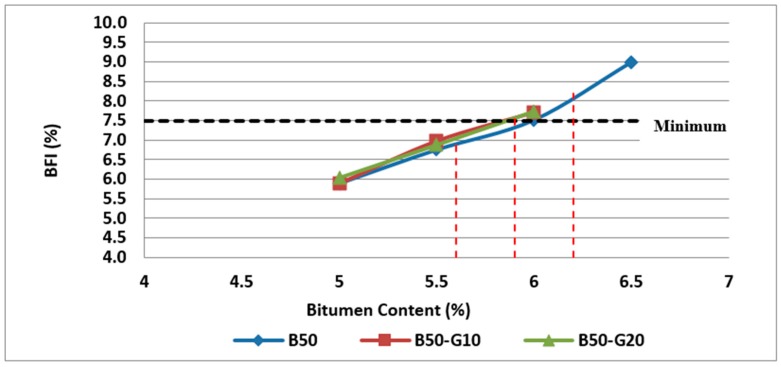
Effect of bitumen content and glass content on BFI of Mix III containing 50% RCA without glass and Mix VI and Mix VII containing 50% RCA as coarse aggregate and glass as fine aggregate.

According to the results obtained, the binder film index of different mixtures were investigated in this research and the variation of BFI with glass and bitumen content in two groups of samples containing 25% and 50% RCA are illustrated in Figure 20 and Figure 21.

As can be observed in Figure 20 and Figure 21, both samples containing glass have slightly higher binder film thickness than samples without glass. However, the comparison of samples at their optimum bitumen content shows that BFI for samples containing 20% glass is less than the minimum requirements of 7.5 microns. 

### 4.5. Volumetric Analysis of Asphalt Mixtures at Optimum Bitumen Content

The results of volumetric properties evaluation of asphalt mixtures with different combinations of aggregates at their optimum content are presented in Table 9. As shown in Table 9, the results of the tests on asphalt mixtures reveal that all mixtures made of RCA meet the standard limits for volumetric properties. However, their high bitumen absorption necessitates the study of volumetric properties of asphalt mixtures made with RCA in combination with recycled glass. To this point, as can be observed in Table 9, all asphalt mixtures containing RCA and 10% recycled glass meet the Australian Standards’ requirements and therefore are deemed appropriate for consideration as asphalt mix design. In addition, results for samples with RCA and 50% of recycled glass, except for binder film index and VMA (which are shown in bold in Table 9), meet the standard typical values. However, VMA for these samples are within the Australian Standards limits. To this point, it can be concluded that asphalt mixtures containing 25% RCA and 10% glass are the most comparable mixtures with control samples. 

In addition, for all samples at different rates of bitumen content, as presented in Table 7 and Table 8, it can be observed that increasing the bitumen content results in the specimen height reduction. This effect can be due to the extra lubrication provided by the hot bitumen during compaction leading to the better and quicker compaction for the same compactive effort.

### 4.6. Resilient Modulus of Asphalt Mixtures at Optimum Bitumen Content

It has been well established that predicting the performance of asphalt mixtures containing recycled materials is very difficult given the inconsistency of materials and their complex interaction with natural materials. Therefore, in this research, primary tests were conducted on different mixtures in terms of materials combinations as well as the materials amount. 

Based on the result of the primary tests, the most acceptable samples were selected for estimating their resilient modulus through the indirect tensile test for three specimens selected based on the results of primary tests, as presented in Table 10. 

**Figure 22:**
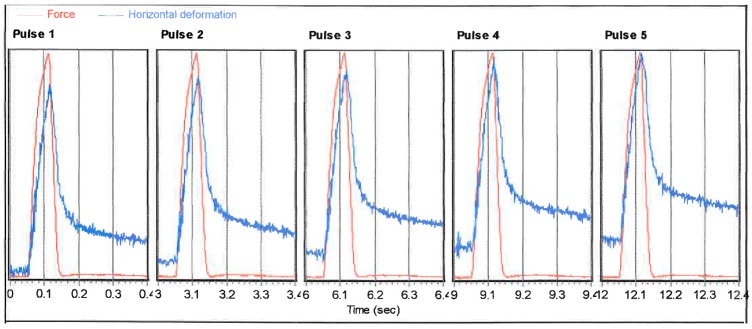
Sample output of Resilient Modulus Test.

To conduct the resilient modulus test, a triaxial repeated loading machine capable of applying 50 kPa of loading was used considering repeated haversine loading. Using this machine, the test sequence can be monitored through the user-friendly program and all test outputs are sent to a desktop computer (Figure 22).

The results of resilient modulus test conducted on three specimens from each asphalt mixture type are presented in Table 11.

As presented in Table 11, the resilient modulus obtained for the sample containing 10% glass is about 15% higher than the measured values for the conventional asphalt mixtures. These results show that the utilization of waste glass and RCA in asphalt mixtures combine the advantages of producing a better asphalt pavement as well as the waste management problem, which subsequently provides a reduction in cost and resources demand.

It should be mentioned that the coefficient of variation is used as an indication to measure the heterogeneity of test results. The results of calculation of standard deviation (SD) and coefficient of variation (COV) for test results provided in Table 11, has shown that the coefficient of variation and standard deviation for the data sets were in an acceptable range of 1.05 to 2.02 (for coefficient of variation) and 0.023 to 0.134 (for standard deviation), revealing that the test results dispersion is low and the tests are conducted consistently.

## 5. Further Research

Although many laboratory and field investigations have been already performed on the performance of asphalt mixtures made with recycled materials such as RCA and recycled glass, more studies are still required to deal with the challenges of this sustainable approach for further use. In this regard, a set of recommendations are provided for researching the engineering properties and other aspects of this technology, as follows:

The incorporation of waste glass in asphalt mixtures affects the bitumen absorption of mixtures, and therefore can compensate for the high bitumen-absorbing RCA. This research investigated the effect of glass on asphalt mixtures containing RCA considering three different percentages. However, it is recommended to investigate the effect of glass size, colours of glass and the glass content in asphalt mixtures.

Further investigation is required on the fatigue behaviour and also the ageing of asphalt mixtures containing RCA and glass.

## 6. Conclusions

This research project was aimed at investigating the feasibility of using recycled glass for compensation of high bitumen absorption of asphalt mixtures containing RCA. The test results on aggregates and asphalt mixtures containing RCA with/without glass indicate that:

(1) RCA has lower flakiness index and misshapen particles compared to basalt implying that asphalt mixtures with a certain amount of RCA can provide better workability, compaction and rutting resistance.
(2) RCA has considerably higher water absorption and wet/dry strength variation in comparison with the virgin aggregate. The results of tests conducted on RCA showed that RCA still meets the standards requirements for aggregates in asphalt mixtures. However, the high water absorption of RCA should be compensated.
(3) Asphalt mixtures containing RCA have a lower bulk density, VMA, VFB and BFI than control mixes, whereas the air voids are higher for mixtures containing RCA. Lower bulk density of RCA will result in cost reduction, as asphalt jobs are mostly measured in cubic meter and materials are purchased in tonnes.
(4) The results of tests on different asphalt mixtures containing different percentages of RCA indicated that RCA increase results in an increase in the optimum bitumen content of the mixtures. Hence, the selection of a proper combination of RCA and other aggregates is required for satisfying the relevant standard requirements.
(5) Utilization of recycled glass with very low water absorption in asphalt mixtures with different combinations of RCA were observed to reduce the bitumen absorption of these asphalt mixtures.
(6) The results of tests on different asphalt mixtures containing RCA and glass indicate that the bitumen absorption decreases with glass increase in asphalt mixtures. In other words, asphalt mixtures containing glass have a lower optimum bitumen content in comparison with asphalt mixtures without glass. So that, as presented in Table 8, asphalt mixtures containing 75% RCA with 10% and 20% glass have optimum bitumen content very close to the optimum content of control samples (asphalt mixtures without any recycled materials).
(7) The results of tests on asphalt mixtures containing RCA and glass at different rate of bitumen content reveals that air void, VMA and bulk density are lower than the corresponding values for asphalt mixtures containing RCA without glass, whereas introducing glass in the asphalt mixtures increases VFB in asphalt mixtures containing both RCA and glass than asphalt mixtures containing only RCA.
(8) The results of volumetric properties of all asphalt mixtures at their optimum bitumen content, as shown in Table 9, indicates that asphalt mixtures made by combining 25% RCA and 10% glass is the most comparable mixture to control samples in terms of volumetric properties and optimum bitumen content requirements.
(9) The results of the resilient modulus test on selected asphalt mixtures reveals that asphalt mixtures made of 25% RCA and 10% glass have about 15% more stiffness than conventional mixtures.
(10) Since the resilient moduli of asphalt mixtures is an important parameter in characterization of the entire structural performance of pavement affecting the layer thickness, the service life and the overall cost of pavement construction, the result of the resilient modulus test indicates that asphalt mixtures made of a combination of 25% RCA and 10% glass will result in the improvement of the structural performance of asphalt pavements as well as the environmental and economic advantages.

## Figures and Tables

**Table 1:** Advantages and disadvantages of utilization of glass in asphalt mixture.

**Advantage**	**Description**
Increased road safety	Since the glass particles have low water absorption, the pavement surface gets dry faster after rain
Easier to compact and cartage over longer distance	Glass asphalt mixtures hold heat longer compared to conventional asphalt mixtures
Improved night time road visibility	Glass asphalt surfaces are more reflective in comparison with conventional asphalt surfaces
Waste reduction	Glass wastes are not disposed into the landfills offering environmental benefits and saving costs
Improved workability	The presence of long and flat particles will positively affect the workability of asphalt mixtures
Commercial benefits	Raw materials are replaced by glass resulting in savings on costs of materials
No need to change asphalt paving process	The same construction method used for conventional asphalt mixtures can be used for asphalt mixtures containing glass
Improved resistance to thermal cracking	The small inflation coefficient of glass improves the thermal cracking resistance
**Disadvantage**	**Description**
Bleeding problem	Low bitumen absorption and density may cause a bleeding problem
Stripping problem	The smooth surface of glass particles reduces the adhesion of asphalt film to the crushed glass, which may cause stripping of the asphalt mixture.
Decreased transverse stability	The angularity and friction angle of glass particles provides inadequate transverse stability, particularly at braking or start-up
Sensitive to water damage	The high silica content in glass particles will make asphalt mixtures made with glass have more moisture sensitivity depending on the glass particle size or the glass content in asphalt mixture
Abrasion of tires	The presence of long and flat particles (particularly in case of large glass particles size) may result in abrasion of tires
Decreased skid resistance	High amount of large size glass particles cause a decrease in skid resistance

**Table 2:** Characteristics of the original bitumen.

Characteristics	Unit	Methods	Value
Softening point	°C	AS 2341.18 [55]	52
Penetration at 25 °C	dmm	AS 2341.12 [56]	min 40
Flashpoint	°C	AS 2341.14 [57]	min 250
Viscosity at 60 °C	Pa·s	AS 2341.2 [58]	320
Viscosity at 135 °C	Pa·s	AS 2341.2 [58]	0.5
Specific Gravity	Kg/m^3^	AS 2341.7 [59]	1.03

**Table 3:** Bitumen and aggregate mix combination rates.

Mix Name	Specimen Name	Coarse Aggregate (%)		Bitumen Content (%)	Fine Aggregate (%)	
		RCA	Basalt		Glass	Basalt
Mix I	B100-4.5	0	100	4.5	0	100
	B100-5	0	100	5	0	100
	B100-5.5	0	100	5.5	0	100
Mix II	B75-5	25	75	5	0	100
	B75-5.5	25	75	5.5	0	100
	B75-6	25	75	6	0	100
Mix III	B50-5	50	50	5	0	100
	B50-5.5	50	50	5.5	0	100
	B50-6	50	50	6	0	100
	B50-6.5	50	50	6.5	0	100
Mix IV	B75-G10-5	25	75	5	10	90
	B75-G10-5.5	25	75	5.5	10	90
	B75-G10-6	25	75	6	10	90
Mix V	B75-G20-5	25	75	5	20	80
	B75-G20-5.5	25	75	5.5	20	80
	B75-G20-6	25	75	6	20	80
Mix VI	B50-G10-5	50	50	5	10	90
	B50-G10-5.5	50	50	5.5	10	90
	B50-G10-6	50	50	6	10	90
Mix VII	B50-G20-5	50	50	5	20	80
	B50-G20-5.5	50	50	5.5	20	80
	B50-G20-6	50	50	6	20	80

**Table 4:** Volumetric parameters requirements for DGA AC14 (AS2150-2005).

Parameter	Range	Typical Values	Description
Air Void	3–6%	5%	Mixtures prepared in accordance with RMS T662
VMA	13–20%	≥15	Mixtures prepared in accordance with RMS T662
VFB	65–80%	-	Mixtures prepared in accordance with RMS T662
Binder Film Index	-	≥7.5 μm	Determined in accordance with Test Method Austroads AG:PT/T237 or AS 2891.8
Filler-Binder Ratio	0.8–1.2	-	Mixtures prepared in accordance with RMS T662

**Table 5:** Test results for evaluation of coarse aggregate properties.

Test	Test Method	Aggregate		Typical Limit Based on Australian Standards
		RCA	Basalt	
Flakiness Index Test	AS 1141.15 [69]	6.9	19.0	25% (max)
Particle Shape Test	AS 1141.14 [70]	6.2	18.3	35% (max)
Water Absorption	AS 1141.6.1 [71]	**6.30**	1.64	2% (max)
Particle Density	AS 1141.6.1 [71]	2.370	2.640	-
Particle Density on Dry Basis	AS 1141.6.1 [71]	2.212	2.530	-
Particle Density on SSD Basis	AS 1141.6.1 [71]	2.351	2.571	-
Aggregate Crushing Value	AS 1141.21 [72]	29.21	8.91	35% (max)
Weak Particles	AS 1141.32 [73]	0.23	0.23	1% (max)
Wet/Dry Strength Test	AS 1141.22 [74]	26.6	8.5	35% (max)
Wet Strength	AS 1141.22 [74]	**119.7**	359.2	150 kN (min)
Dry Strength	AS 1141.22 [74]	163.1	392.9	-

**Table 6:** Test results for evaluation of fine aggregate properties.

Test	Test Method	Aggregate		Typical Limit Based on Australian Standards
		Glass	Basalt	
Water Absorption	AS 1141.5 [75]	0.10	2.35	3% (max)
Particle Density	AS 1141.5 [75]	2.489	2.879	-
Particle Density on Dry Basis	AS 1141.5 [75]	2.483	2.610	-
Particle Density on SSD Basis	AS 1141.5 [75]	2.485	2.668	-

**Table 7:** Volumetric properties of asphalt mixtures.

Specimen Name	AV (%)	Water Absorption (%)	Bulk Density (gr/cm^3^)	VMA (%)	VFB (%)	Binder Film Index (µm)	Filler-Binder Ratio	Height (mm)
B100-4.5	7.2	0.46	2.398	16.4	59.6	6.9	1.2	69.0
B100-5	5.4	0.17	2.439	15.4	69.0	7.5	1.1	67.0
B100-5.5	4.1	0.11	2.441	15.8	78.6	8.7	1.0	66.3
B75-5	6.7	0.43	2.398	15.3	59.7	6.4	1.1	69.7
B75-5.5	5.4	0.23	2.410	15.3	68.7	7.4	1.0	67.6
B75-6	4.8	0.15	2.411	15.7	73.8	8.2	0.9	67.0
B50-5	7.3	0.56	2.355	15.3	55.3	5.9	1.1	73.6
B50-5.5	6.0	0.31	2.371	15.1	64.0	6.8	1.0	71.0
B50-6	5.4	0.24	2.373	15.5	69.1	7.5	0.9	68.9
B50-6.5	4.5	0.16	2.359	16.5	77.1	9.0	0.9	68.2

**Table 8:** Volumetric properties of asphalt mixtures containing RCA and glass.

Specimen Name	AV (%)	Water Absorption (%)	Bulk Density (gr/cm^3^)	VMA (%)	VFB (%)	Binder Film Index (µm)	Filler-Binder Ratio	Height (mm)
B75-G10-5	5.9	0.29	2.394	14.8	63.9	6.6	1.1	68.2
B75-G10-5.5	4.7	0.16	2.400	15.1	73.0	7.7	1.0	67.1
B75-G10-6	4.3	0.14	2.406	15.3	76.3	8.2	0.9	66.1
B75-G20-5	5.6	0.27	2.384	14.5	65.2	6.6	1.1	68.1
B75-G20-5.5	4.6	0.15	2.394	14.6	72.7	7.4	1.0	67.8
B75-G20-6	4.2	0.12	2.398	14.9	76.3	7.9	0.9	65.7
B50-G10-5	6.9	0.54	2.352	14.9	56.9	5.9	1.1	69.9
B50-G10-5.5	5.5	0.27	2.363	14.9	67.0	7.0	1.0	69.7
B50-G10-6	4.9	0.18	2.366	15.3	72.1	7.7	0.9	69.0
B50-G20-5	6.5	0.30	2.339	14.7	59.2	6.0	1.1	69.4
B50-G20-5.5	5.3	0.21	2.353	14.6	67.7	6.9	1.0	68.6
B50-G20-6	4.2	0.14	2.364	14.7	75.8	7.7	0.9	67.7

**Table 9:** Volumetric properties of asphalt mixtures at optimum bitumen content.

Specimen Name	Optimum Bitumen Content (%)	Bulk Density (gr/cm^3^)	VMA (%)	VFB (%)	Binder Film Index (µm)	Filler-Binder Ratio
B100	5.1	2.442	15.5	71.5	7.8	1.1
B75	5.8	2.411	15.6	72.0	7.9	0.9
B75-G10	5.4	2.398	15.0	70.5	7.5	1.0
B75-G20	5.3	2.390	**14.5**	69.5	**7.1**	1.0
B50	6.2	2.368	15.9	72.2	8.2	0.9
B50-G10	5.9	2.365	15.2	70.8	7.6	0.9
B50-G20	5.6	2.355	**14.6**	69.5	**7.1**	1.0
**Standard Limit**	-	-	13–20%	60–80%	-	0.8–1.2
**Typical Value**	-	-	15% (min)		7.5 µm (min)	-

**Table 10:** Material combination for preparation of specimens of indirect tensile test.

Asphalt Mixture	Coarse Aggregate (%)		Fine Aggregate (%)		Optimum Bitumen Content (OBC, %)
	Basalt	RCA	Basalt	Glass	
B100	100	0	100	0	5.1
B75	75	25	100	0	5.8
B75-G10	75	25	90	10	5.4

**Table 11:** Result of resilient modulus test in accordance with AS 2891.13.1 (2013).

Asphalt Mixture	Ave. Sample Height (mm)	Ave. Sample Diameter (mm)	Peak Load (N)	Recovered Horizontal Strain (µɛ)	$M_{r}$ (MPa)
B100	64.95	99.925	2718.7	50.01	5613
B75	68.225	99.95	3252.7	51.52	6205
B75-G10	68.025	99.975	3302.6	49.08	6632
